# Supplementary material for: Autophagy is a gatekeeper of hepatic differentiation and carcinogenesis by controlling the degradation of Yap
Source: Nat Commun. 2018 Nov 23;9:4962. doi: 10.1038/s41467-018-07338-z (PMC6251897; doi:10.1038/s41467-018-07338-z)
Supplement: Supplementary file 1 — Supplementary Information [file 41467_2018_7338_MOESM1_ESM.pdf]

**Supplementary Information**

# Autophagy is a Gatekeeper of Hepatic Differentiation and Carcinogenesis by Controlling the Degradation of Yap

**Lee et al.**

## Supplementary Figure 1

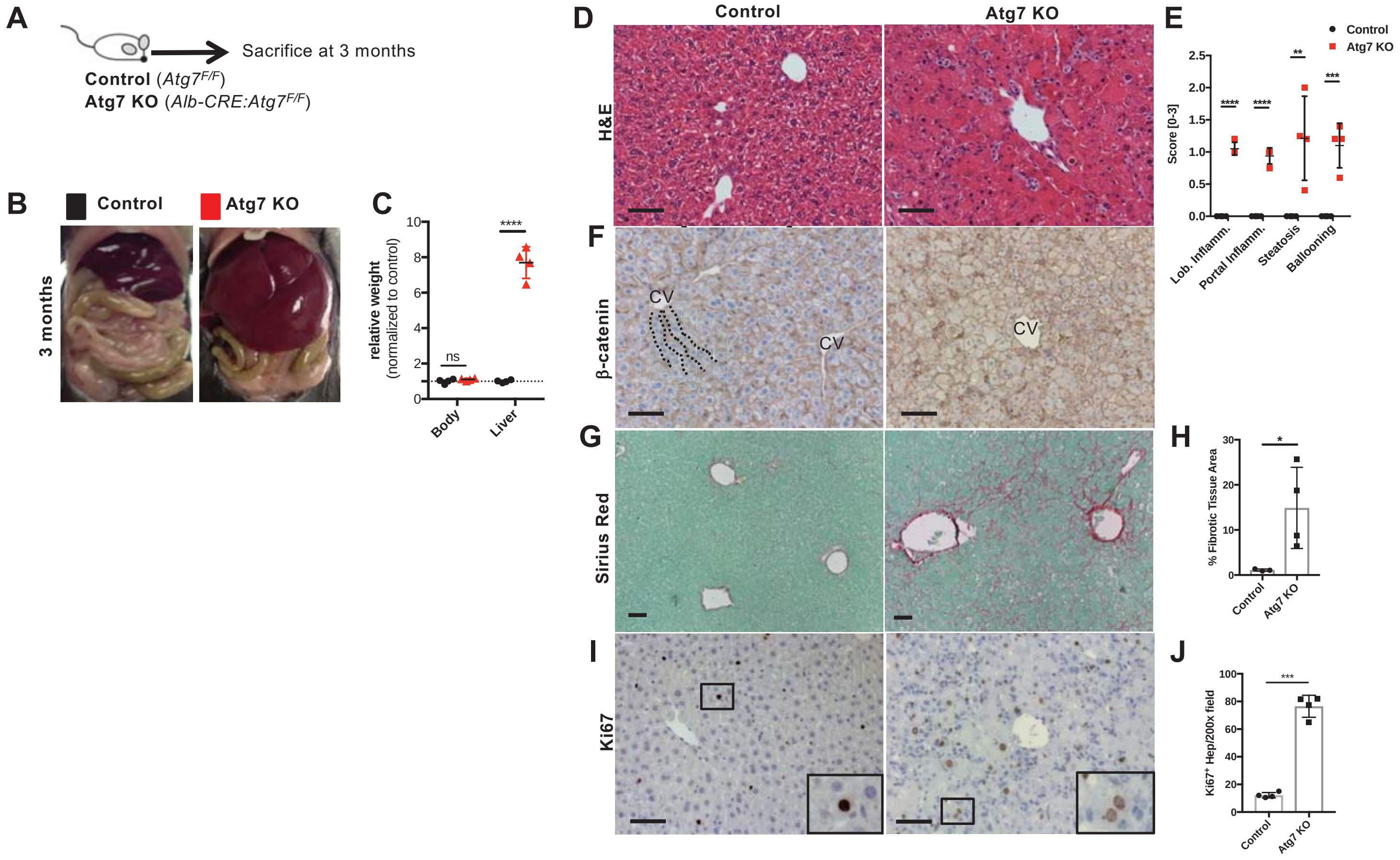


**Supplementary Figure 1 | Autophagy maintains organ size, cell size and number, differentiation in hepatocytes.**

A. Control (Atg7$^{F/F}$) and Atg7 KO mice (Alb-CRE:Atg7$^{F/F}$) were analyzed at 3 months of age, n=4 per group.
B. Representative images of gross liver morphology of control and Atg7 KO mice
C. Relative body and liver weights of control and Atg7 KO animals. ns, not significant.
D. H&E staining of liver sections from control (Atg7$^{F/F}$) and Atg7 KO (Alb-CRE:Atg7$^{F/F}$) mice.
E. Histological scoring for lobular inflammation, portal inflammation, steatosis and ballooning.
F. Immunostaining for b-catenin in control and Atg7 KO mice. Dashed lines marks some hepatocyte trabeculae.
G. Sirius red staining for fibrotic tissue in control and Atg7 KO mice.
H. Quantification of fibrotic tissue area from sirius red/fast green staining (G) by Bioquant software of control and Atg7 KO liver sections. Evaluation of 25 100x fields per mouse. *P=0.04
I. Immunostaining for Ki67 in control and Atg7 KO mice. Large insert present magnification of small insets.
J. Quantification of Ki67$^+$ nuclei per 200x field per mouse. Ten 200x fields per mouse were analyzed. ***P<0.0001

## Supplementary Figure 2

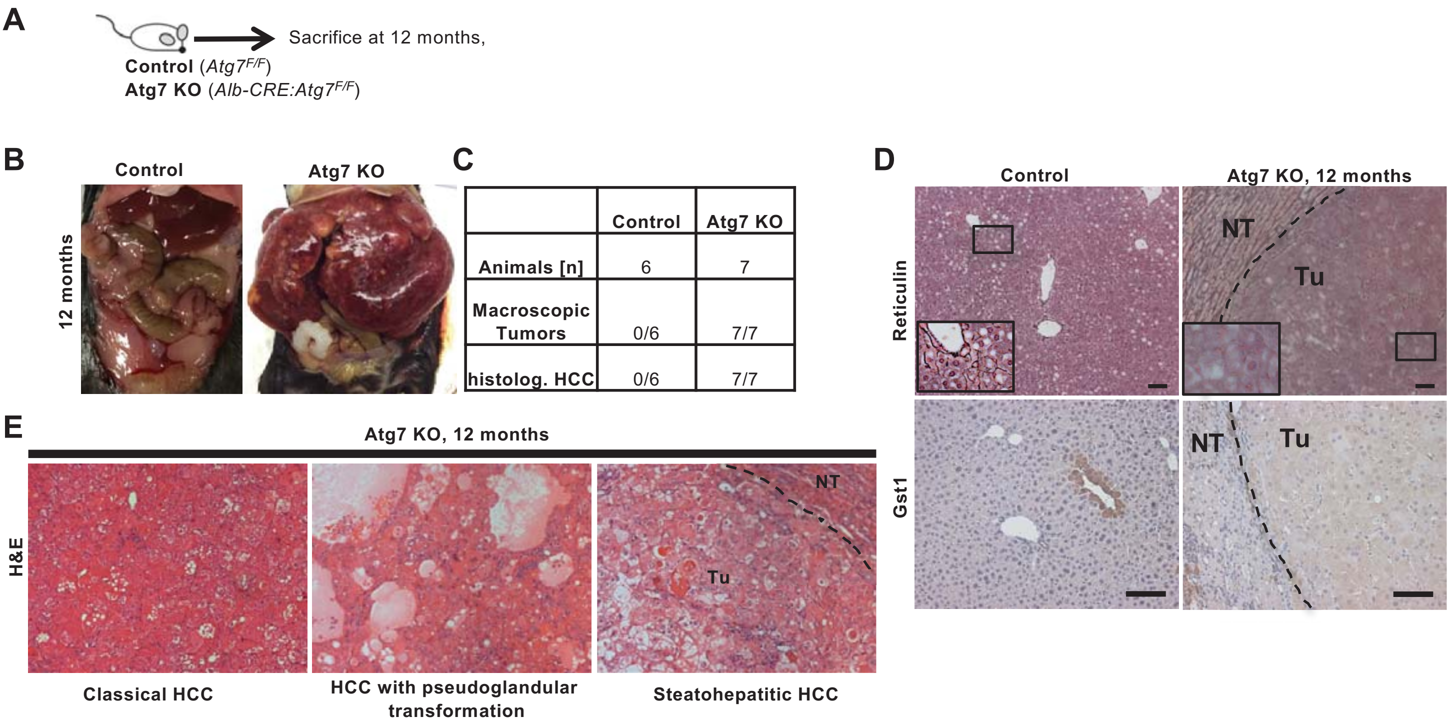


**Supplementary Figure 2 | Autophagy-defective murine livers develop hepatocellular carcinoma.**

A. Control (*Atg7*$^{F/F}$) and Atg7 KO mice (*Alb-CRE:Atg7*$^{F/F}$) were analyzed at 12 months of age, n=6 and 7 respectively.
B. Representative images of gross liver morphology of control and Atg7 KO mice
C. Distribution of control and Atg7 KO animals at 12 months with macroscopic presence or absence of tumors as well as histological HCC diagnosis. HCC, hepatocellular cancer.
D. Reticulin and Gst1 staining of liver sections from control and tumor sections.
E. H&E staining of representative liver tumor sections from Atg7 KO mice.

NT, non-tumorous tissue; Tu, Tumor. Large insert present magnification of small insets. Scale bar indicates 100 µm.

## Supplementary Figure 3

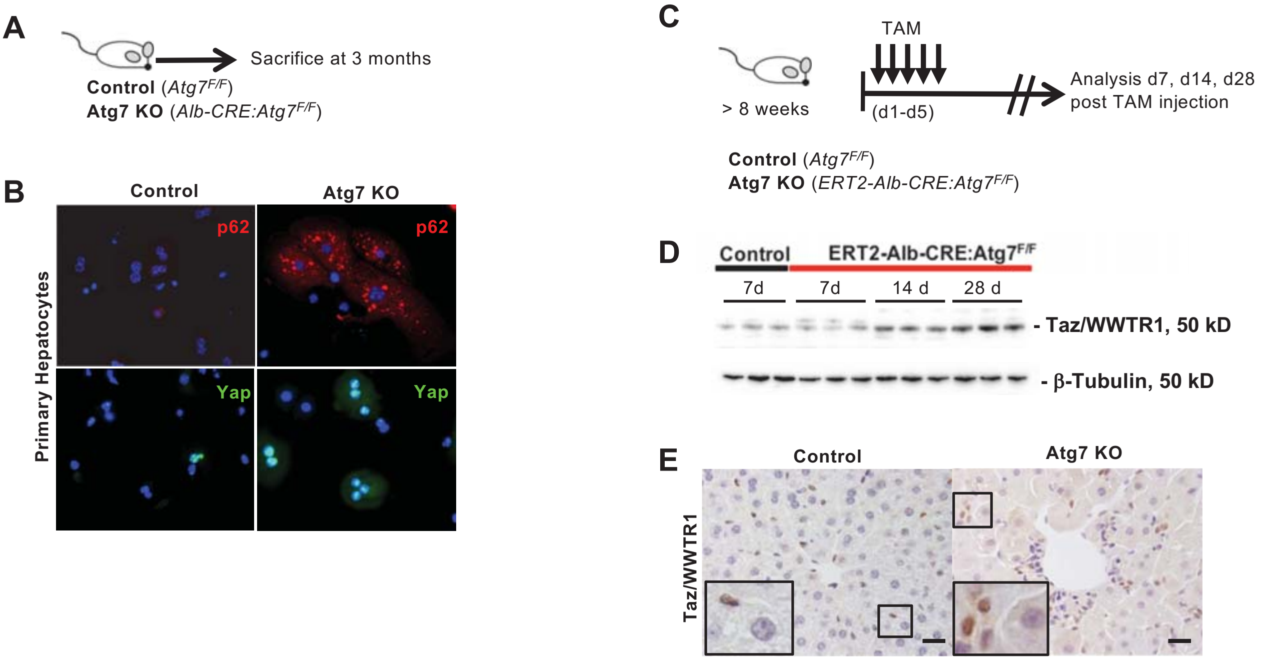


**Supplementary Figure 3 | Autophagy-defective murine livers display increased expression of Hippo tumor suppressor pathway effectors.**

A. Control (*Atg7*$^{F/F}$) and Atg7 KO mice (*Alb-CRE:Atg7*$^{F/F}$) were analyzed at 3 months of age.

B. Immunofluorescence analysis for p62/Sqstm1 and Yap of primary hepatocytes from control and Atg7 KO mice.

C. Tamoxifen (TAM)-inducible, hepatocyte-specific Atg7 KO (*ERT2-Alb-CRE:Atg7*$^{F/F}$) and respective controls were analyzed 7 days, 14 days and 28 days after TAM injection. TAM, tamoxifen.

D. Immunoblotting of whole liver lysates from controls and Atg7 KO mice for Taz/Wwtr1 and b-tubulin 7 days, 14 days and 28 days after TAM injection.

E. Immunohistochemistry for Taz/Wwtr1 in Atg7 KO mice (Alb-CRE/Atg7$^{F/F}$, 3 months of age). Large insets present magnification of small insets. Scale bar indicate 100 µm.

## Supplementary Figure 4

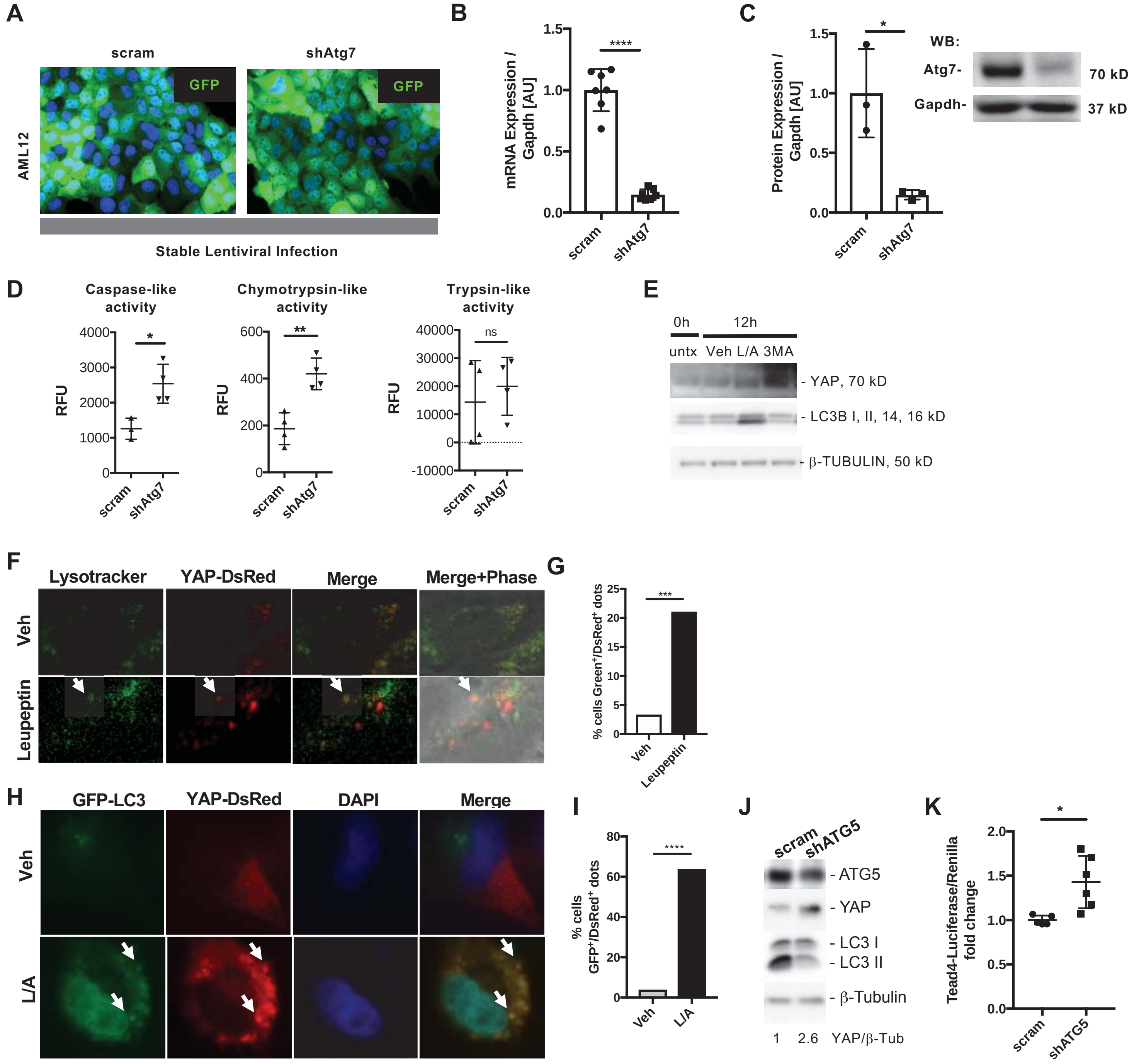


**Supplementary Figure 4 | Effective knock down of *Atg7* in shAtg7-AML12 cells and lysosomal degradation of Yap.**

A. AML12 cells stably infected with scrambled (scram) or shAtg7 lentiviral vectors expressing GFP.
B. qRT-PCR analysis for *Atg7* mRNA in scram- and in shAtg7-infected AML12 cells. Data from 3 independent experiments, normalized to *Gapdh* expression, mean ± SD. AU, arbitrary units. ****P<0.0001 by two-tailed t-test.
C. Immunoblot analysis for Atg7 in scram and shAtg7-AML12. Quantification by densitometry by normalization to Gapdh expression. Data from 3 independent experiments, mean±SD. * P=0.0167 by two-tailed t-test.
D. Proteasomal activity determination in scram- and shAtg7-AML12 cells. 4 biological replicates per group data representative of 2 independent experiments, mean±SD. *P<0.05, **P<0.005 by two tailed t-test. RFU, relative fluorescent units.

*(Continued on next page)*

**Supplementary Figure 4 | Effective knock down of *Atg7* in shAtg7-AML12 cells and lysosomal degradation of Yap *(continued)***

E. Immunoblotting of whole cell lysates of THLE5B cells incubated with Leupeptin/$NH_4Cl$ or vehicle. Untx, untreated; Veh, vehicle; L/A, Leupeptin/$NH_4Cl$; 3MA, 3-Methyladenine; h, hours.
F. Confocal live imaging analysis of THLE5B cells incubated with LysoTracker Green and transfected with YAP-DsRed. Treatment with Leupeptin or vehicle.
G. Quantitative analysis of cells with LysoTracker Green$^+$ and DsRed$^+$ dots. (n= 89 and 57 cells, respectively). ***P=0.0005 by two tailed t-test.
H. Immunofluorescence analysis of THLEB5 cells transfected with GFP-LC3 and YAP-DsRed. Incubation with Leupeptin/$NH_4Cl$ or vehicle. Veh, vehicle; L/A, Leupeptin/$NH_4Cl$.
I. Quantitative analysis of cells with GFP$^+$/DsRed$^+$ dots. (n= 51 and 69 cells, respectively). ****P<0.0001 by two tailed t-test.
J. Immunoblot analysis of scram- and shATG5 infected THLE5B cell lysates for ATG5, YAP, LC3 and β-tubulin.
K. Tead4-Luciferase analysis in scram- and shATG5 infected THLE5B cells. Data represent mean±SD from 2 independent experiments. *P=0.01 by two tailed t-test.

## Supplementary Figure 5

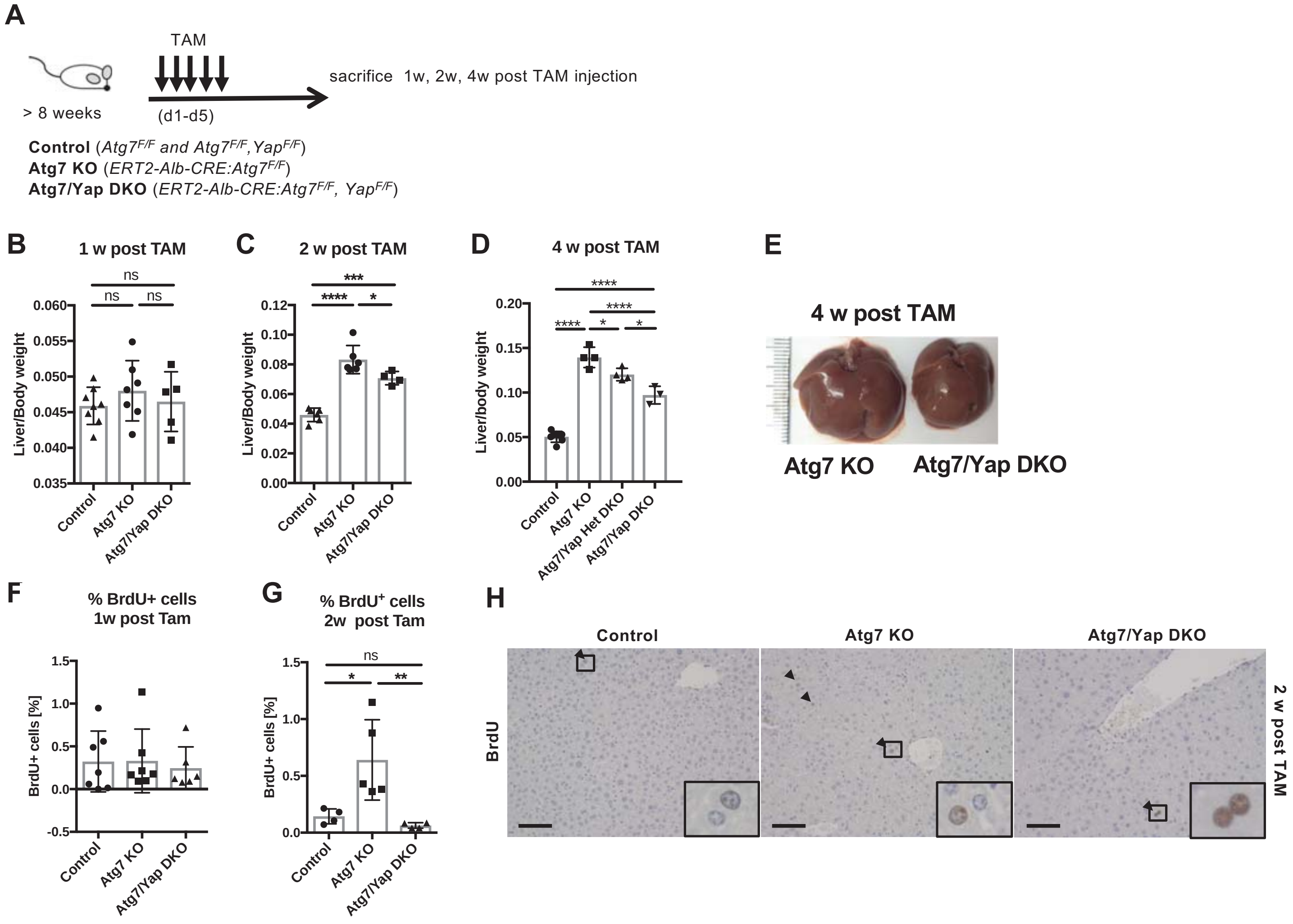


**Supplementary Figure 5 | Yap deletion in Atg7 KO mice attenuates hepatomegaly and proliferative activity in Atg7 KO mice.**

A. Control *Atg7F/F and Atg7F/F,YapF/F*) and TAM-inducible, hepatocyte specific Atg7 KO (*ERT2-Alb-CRE:Atg7F/F*) and Atg7/Yap DKO (*ERT2-Alb-CRE:Atg7F/F, YapF/F*) were analyzed 1 week, 2 weeks, 4 weeks after TAM injection. TAM, tamoxifen.
B. Liver/body weight ratio in control, Atg7 KO and Atg7/Yap DKO mice 1 week after TAM injection. N=4 per group.
C. Liver/body weight ratio in control, Atg7 KO and Atg7/Yap DKO mice 2 weeks after TAM injection. N=4 per group.
D. Liver/body weight ratio in control, Atg7 KO and Atg7/Yap DKO mice 4 weeks after TAM injection. N= 4, 4, 3 animals per group.
E. Representative image of gross liver morphology of Atg7 KO and Atg7/Yap DKO 4 weeks post TAM injection.
F. Quantification of % BrdU$^+$ nuclei per 100x field in liver sections from control, Atg7 KO and Atg7/Yap DKO mice 1 weeks after TAM injection. Ten 100x fields per mouse were analyzed. n=4 per group.
G. Quantification of % BrdU$^+$ nuclei per 100x field in liver sections from control, Atg7 KO and Atg7/Yap DKO mice 2 weeks after TAM injection. Ten 100x fields per mouse were analyzed. n=4 per group.
H. BrdU staining of control, Atg7 KO and Atg7/Yap DKO liver sections. Scale bar 100 µm. Large insets present magnification of small insets.
I. Data represent mean±SD. P-values analyzed by one-way ANOVA and Tukey's HSD. *P<0.05, **P<0.01, ***P<0.0005, ****P<0.0001 unless indicated otherwise. ns, not significant.

## Supplementary Figure 6

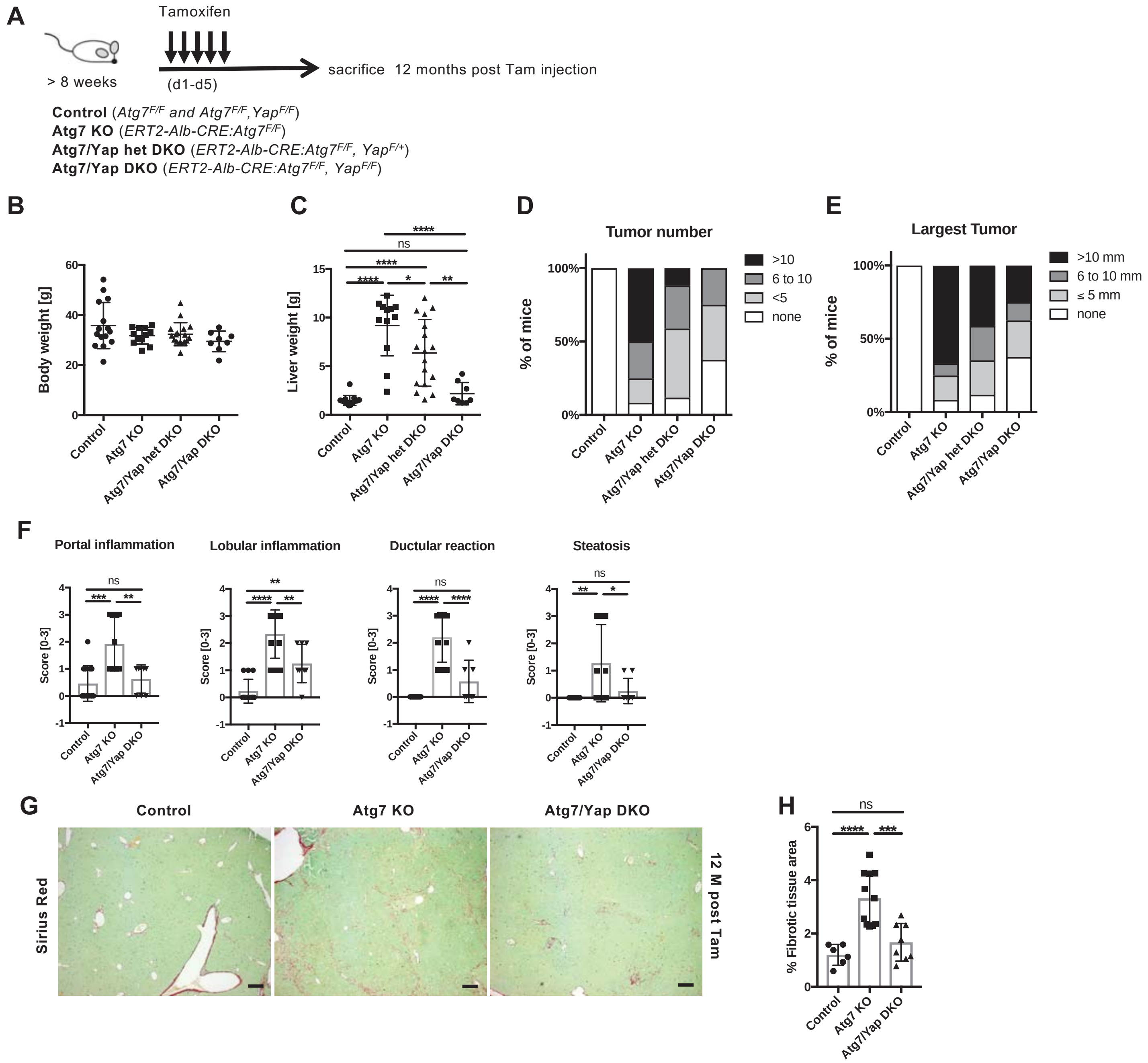


**Supplementary Figure 6 | Loss of Yap in Atg7 KO mice leads to attenuation of liver injury, fibrosis and hepatocarcinogenesis.**

A. Control (*Atg7$^{F/F}$ and Atg7$^{F/F}$, Yap$^{F/F}$*) and TAM-inducible, hepatocyte specific Atg7 KO (*ERT2-Alb-CRE:Atg7$^{F/F}$* ) and Atg7/Yap het DKO (*ERT2-Alb-CRE:Atg7$^{F/F}$, Yap$^{F/+}$)* and Atg7/Yap DKO (*ERT2-Alb-CRE:Atg7$^{F/F}$, Yap$^{F/F}$)* were analyzed 12 months after TAM injection. N=15, 12, 17 and 8 animals, respectively.
B. Body weights of control, Atg7 KO, Atg7/Yap het DKO and Atg7/Yap DKO 12 m post TAM. No significant differences between groups.
C. Liver weights of control, Atg7 KO, Atg7/Yap het DKO and Atg7/Yap DKO 12 m post TAM. **P=0.0018
D. Percentage of mice (control, Atg7 KO, Atg7/Yap het DKO and Atg7/Yap DKO 12 m post TAM) with none, <5, 6-10 or >10 macroscopic tumors.
E. Percentage of mice (control, Atg7 KO, Atg7/Yap het DKO and Atg7/Yap DKO 12 m post TAM) with no tumor or largest tumor size < 5 mm, 6-10 mm or >10 mm.

*(Continued on next page)*

**Supplementary Figure 6 | Loss of Yap in Atg7 KO mice leads to attenuation of liver injury, fibrosis and hepatocarcinogenesis *(continued)***

F. Histological scoring of portal inflammation, lobular inflammation, ductular reaction, steatosis.
G. Sirius red staining of liver section of control, Atg7 KO and Atg7/Yap DKO.
H. Quantification of fibrotic tissue area in control, Atg7 KO and Atg7/Yap DKO. 25 100x pictures per animal were analyzed.

Data represent mean±SD. P-values analyzed by one-way ANOVA and Tukey's HSD. *p<0.05, **p<0.01, ***p<0.001, ****p<0.0001 unless indicated otherwise. ns, not significant. Scale bar indicates 100 μm.

## Supplementary Information – uncropped Immunoblots

### IB Figure 1J

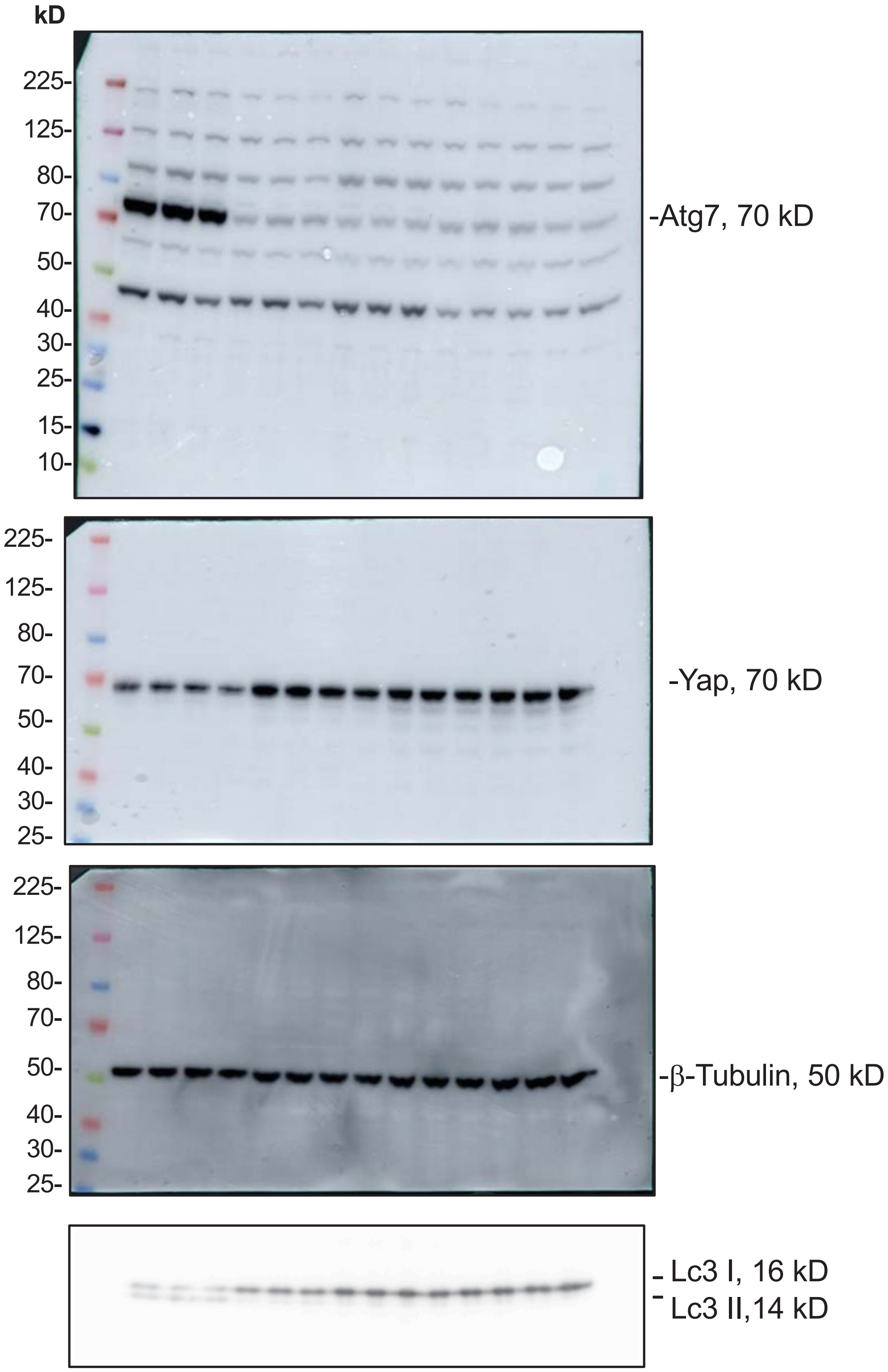

Supplementary Information – uncropped Immunoblots

IB Figure 1J continued

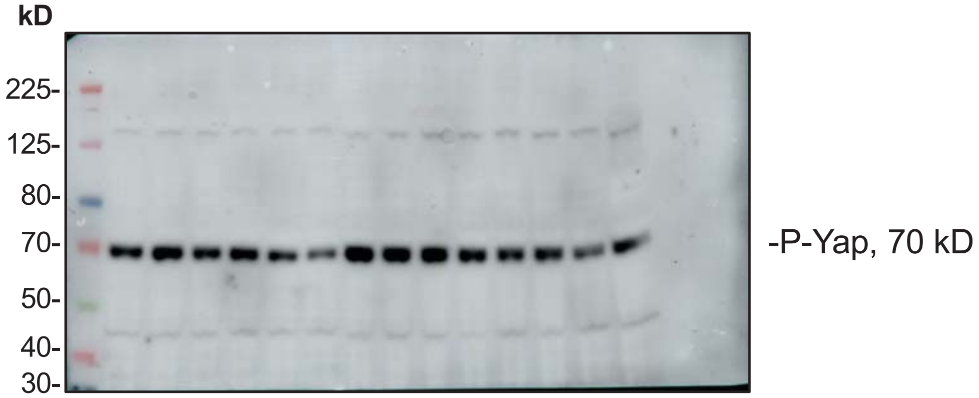


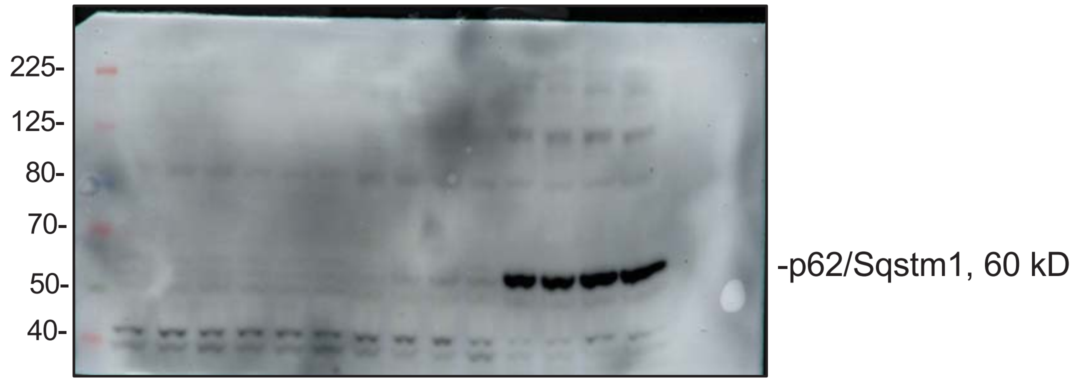

## Supplementary Information – uncropped Immunoblots

### IB Figure 2B

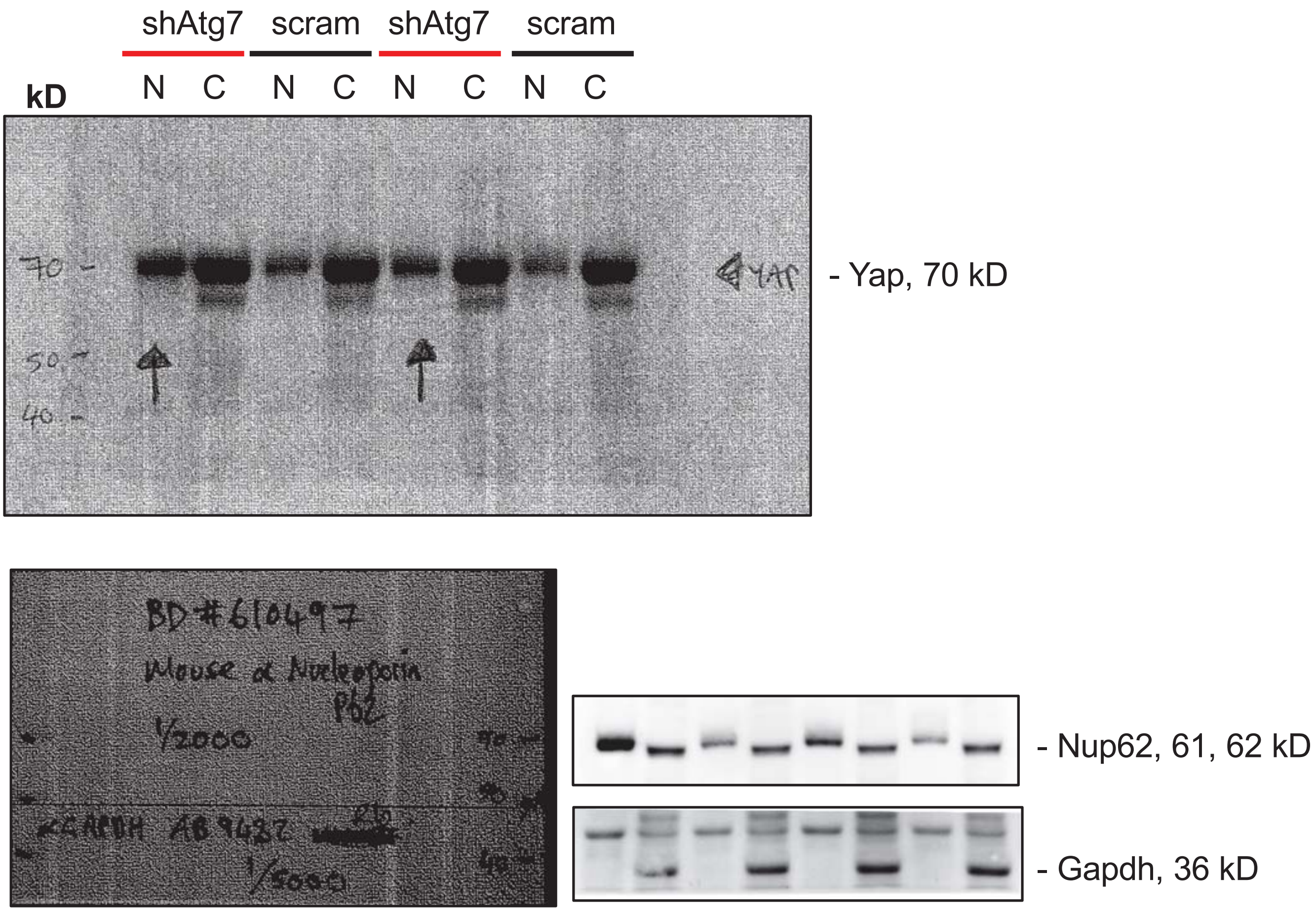

**Supplementary Information – uncropped Immunoblots**

**IB Figure 2E**

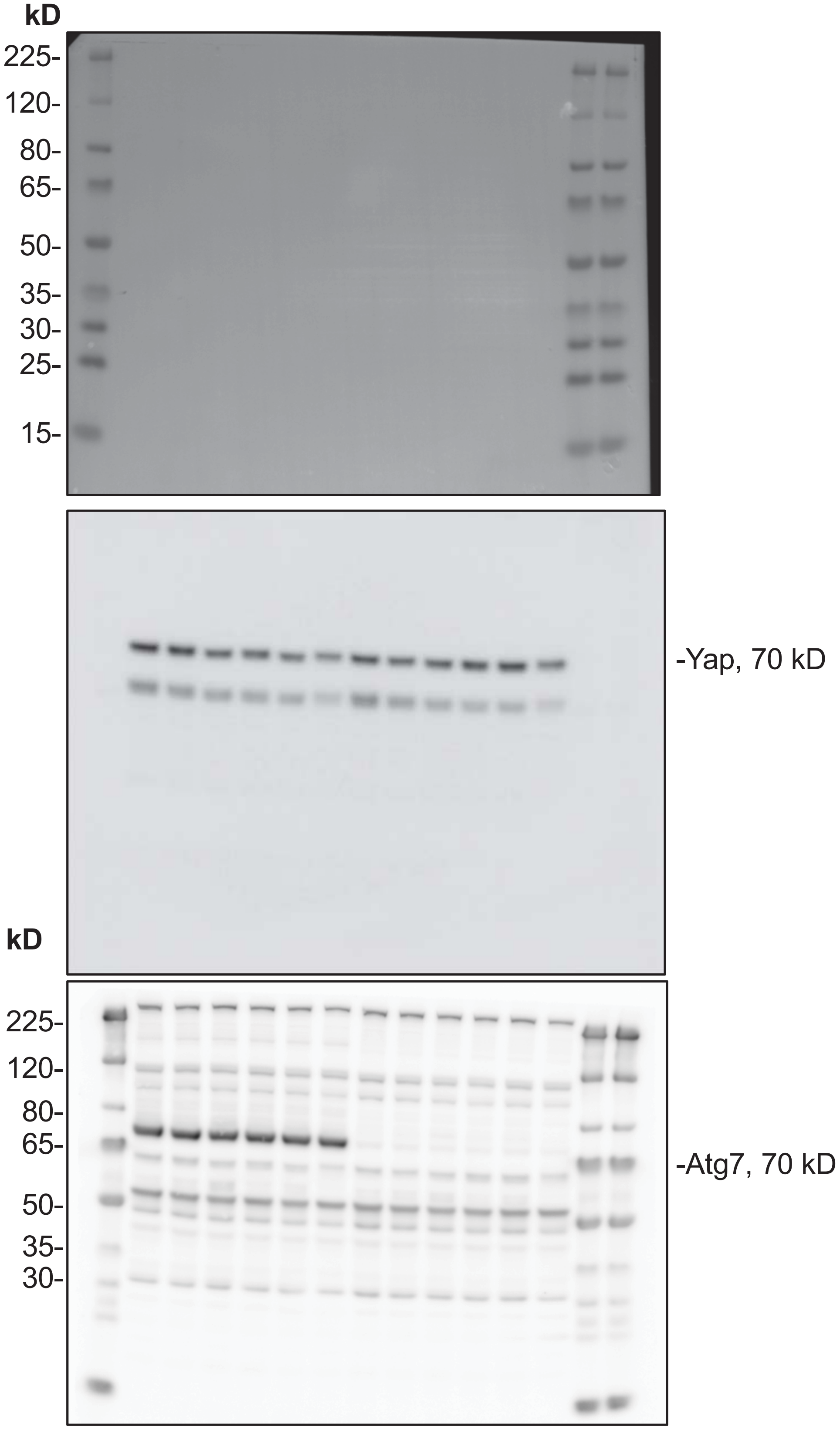

**Supplementary Information – uncropped Immunoblots**

**IB Figure 2E continued**

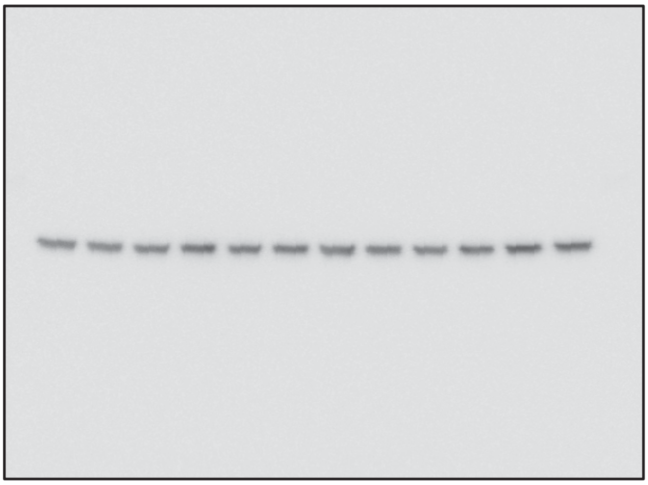

-β-Tubulin, 50 kD

**Supplementary Information – uncropped Immunoblots**

**IB Figure 2G**

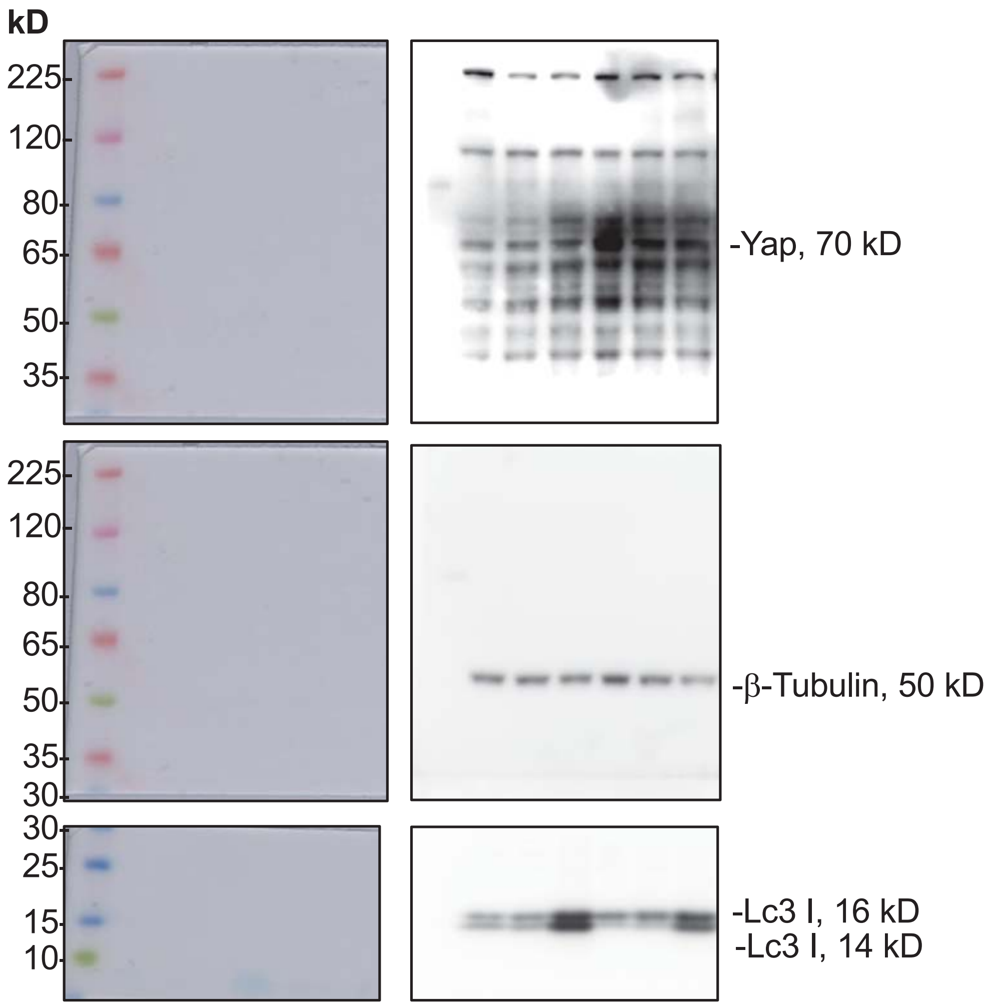

**Supplementary Information – uncropped Immunoblots**

**IB Figure 4 B**

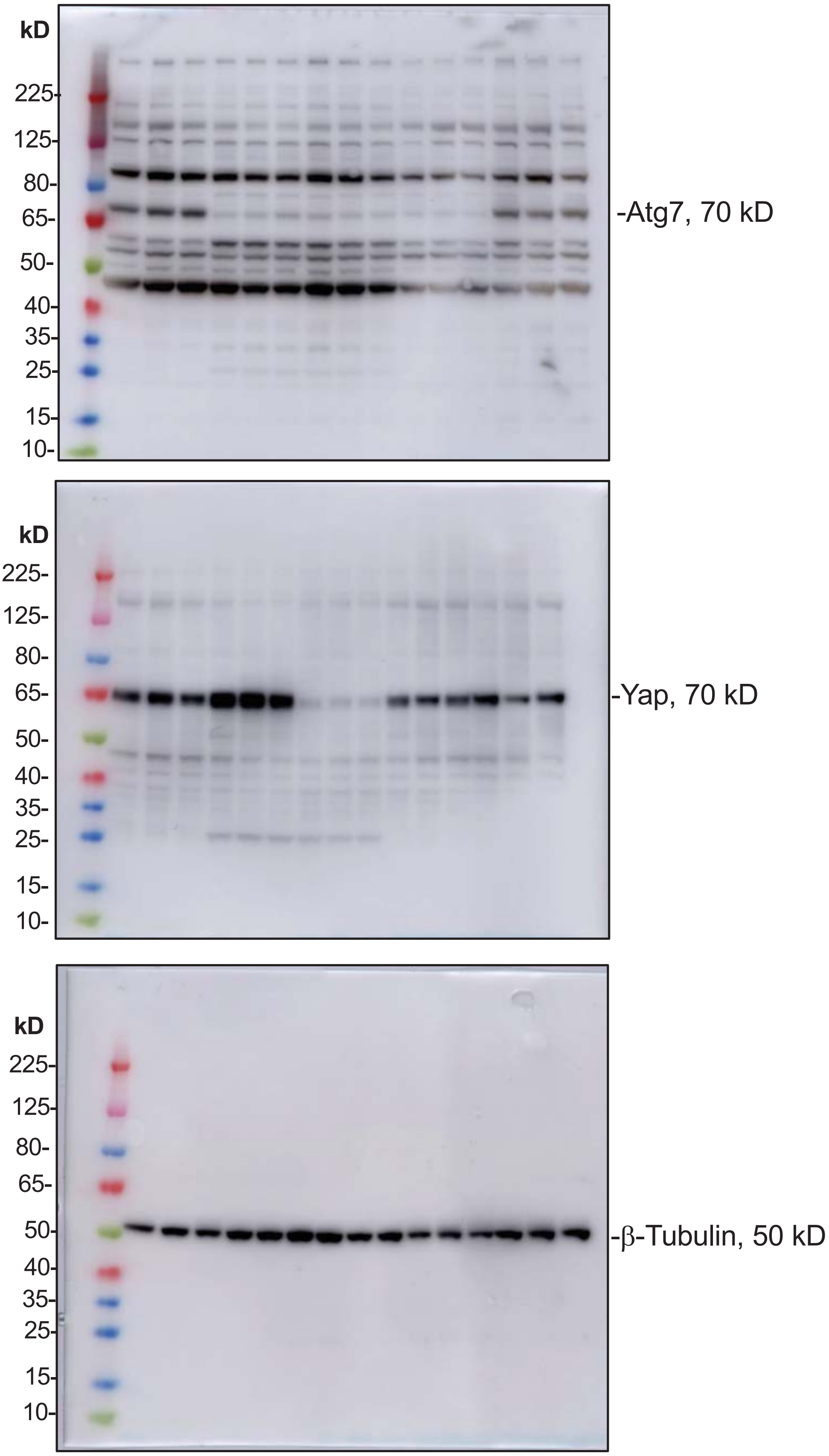

## Supplementary Information – uncropped Immunoblots

### IB Figure 4 B

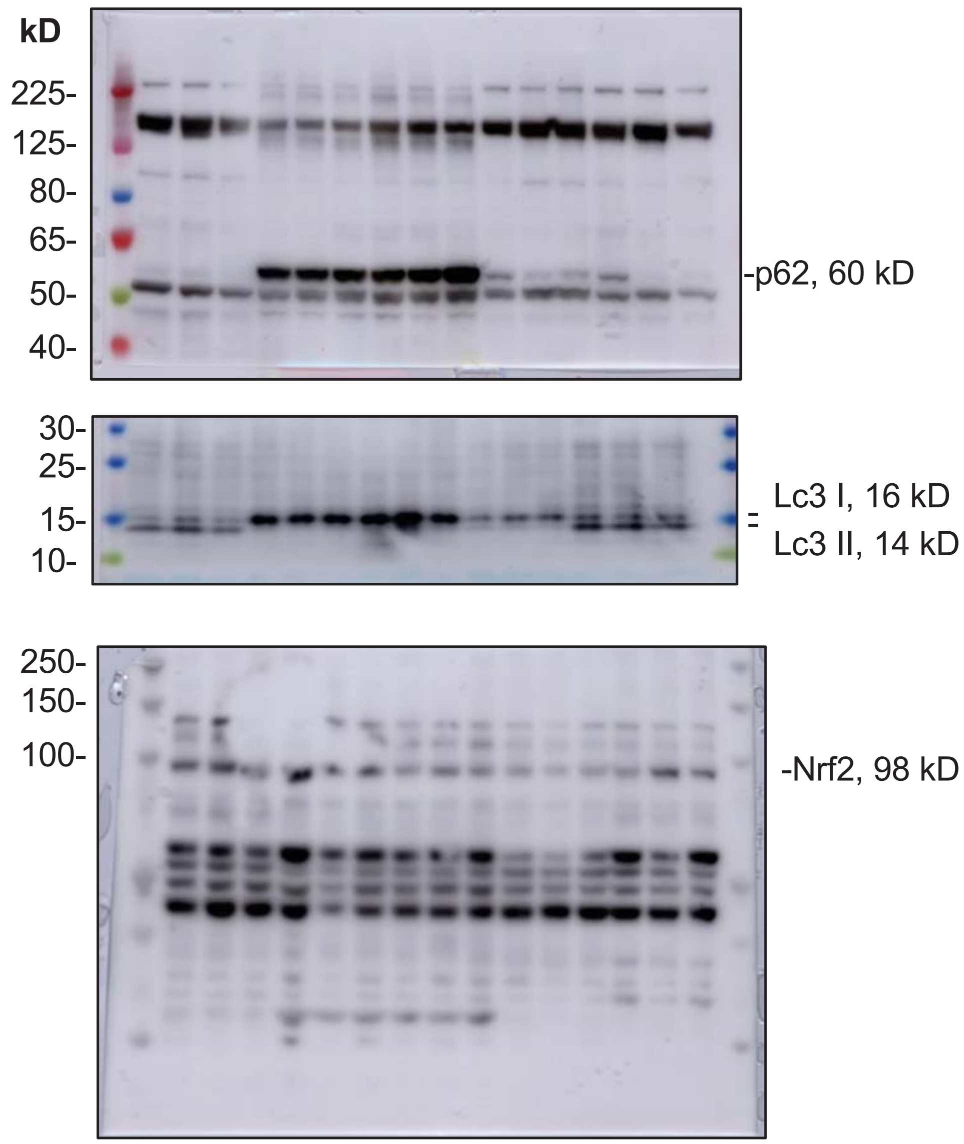

**Supplementary Information – uncropped Immunoblots**

**IB Supplementary Fig. 3D**

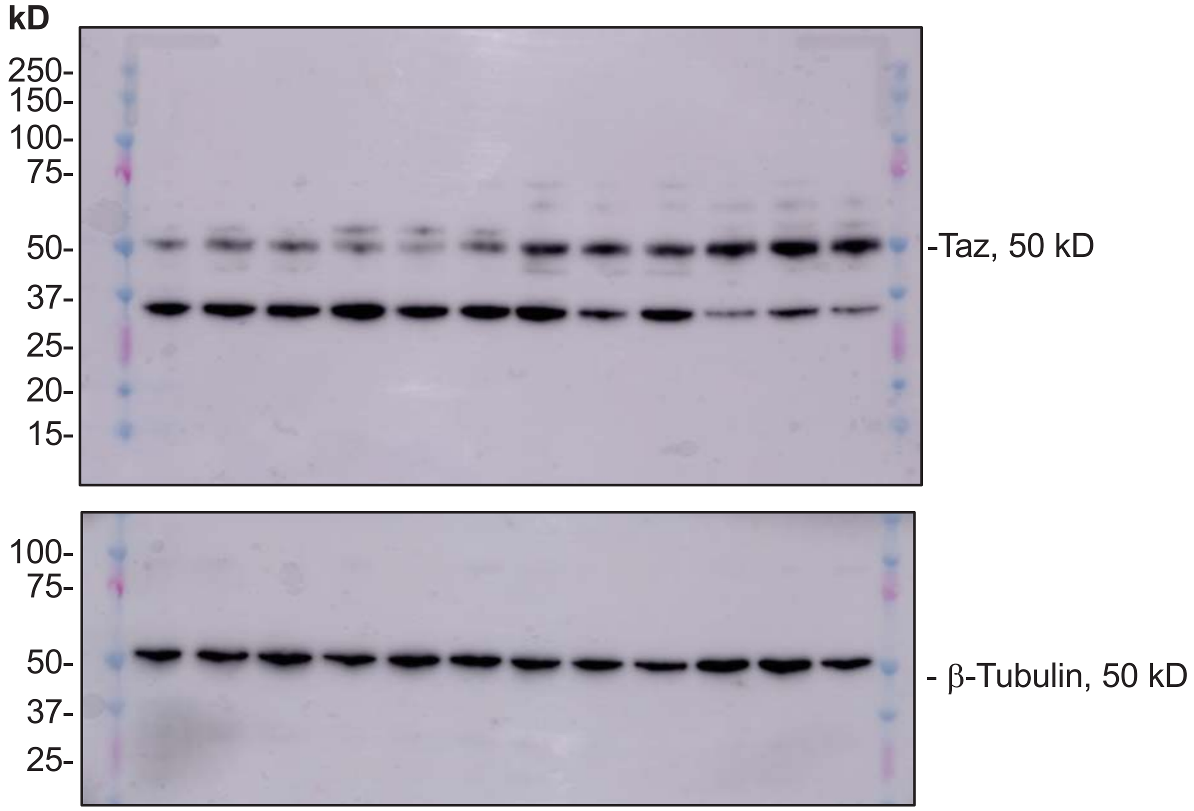

**Supplementary Information – uncropped Immunoblots**

**IB Supplementary Fig. 4C**

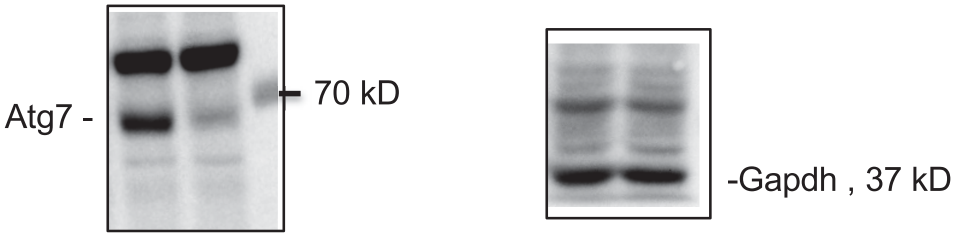


**IB Supplementary Fig. 4E**

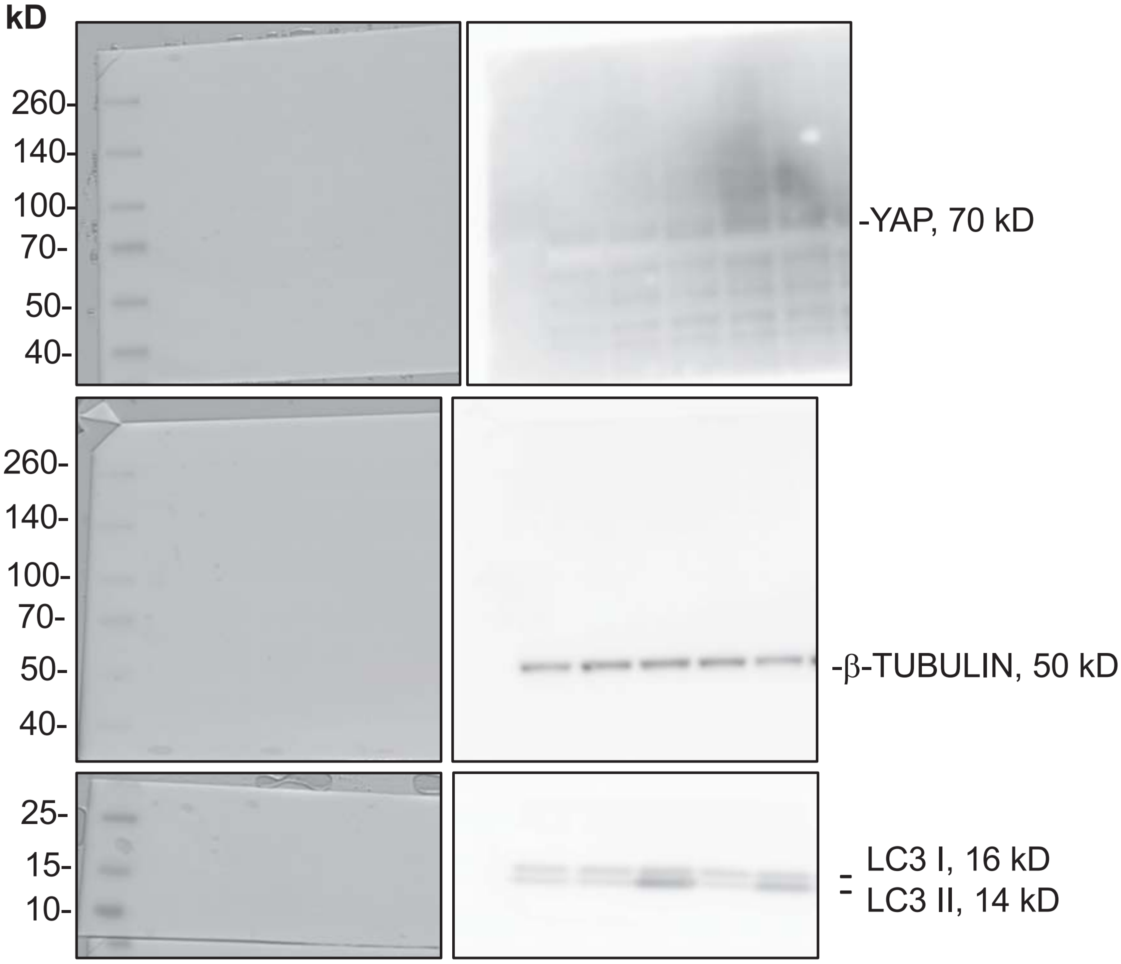

## Supplementary Information – uncropped Immunoblots

### IB Supplementary Fig. 4J

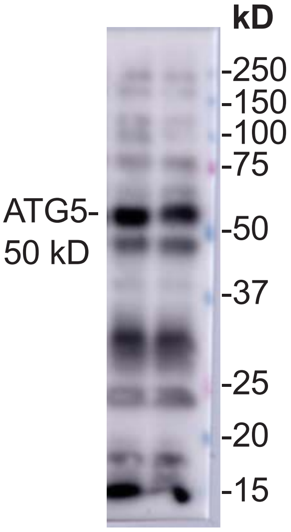


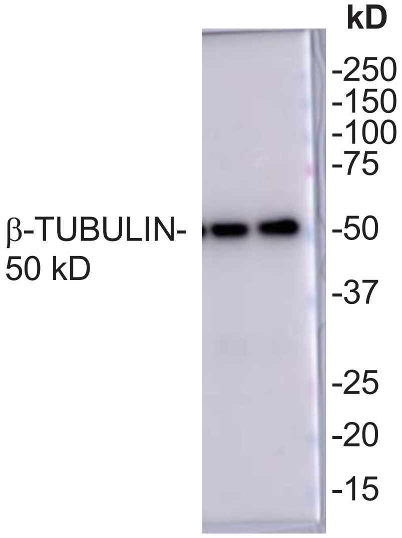


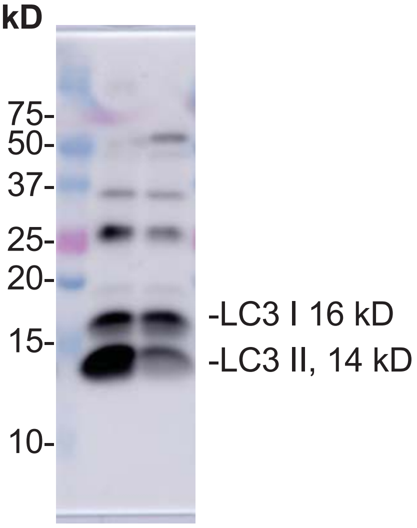


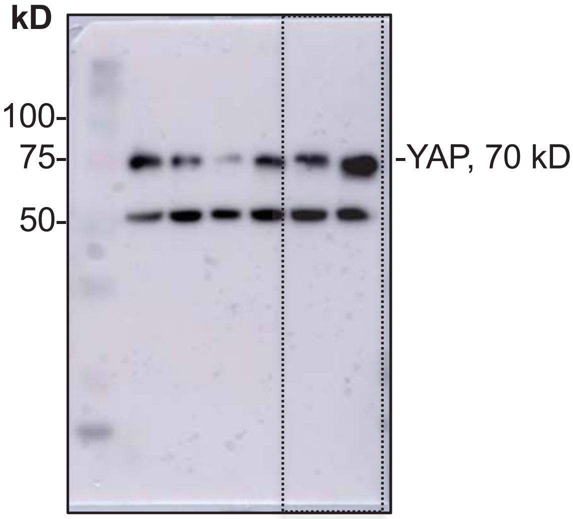

**Supplementary Table 1. Antibodies for Immunohistochemistry and Immunoblotting.**

IB, immunoblotting; IHC, immunohistochemistry; IF, immunofluorescence.

| **Antibody** | **species** | **application** | **dilution** | **cat. Number** | **manufacturer** |
|---|---|---|---|---|---|
| Yap | rabbit | IB | 1:1000 | 4912 | cell signaling |
| | | IHC | 1:500 | | |
| Yap | rabbit | IB | 1:1000 | 14074 | cell signaling |
| | | IHC | 1:500 | | |
| Taz | rabbit | IB | 1:1000 | HPA007415 | Sigma |
| | | IHC | 1:500 | | |
| Ki67 | rabbit | IHC | 1:10000 | 15580 | abcam |
| p62/SQSTM1 | rabbit | IB | 1:4000 | PM045 | MBL |
| | | IHC | 1:4000 | | |
| Atg7 | rabbit | IB | 1:1000 | A2856 | Sigma |
| Yap/Taz | rabbit | IB | 1:1000 | 8418 | cell signaling |
| P-Yap (S127) | rabbit | IB | 1:1000 | 4911 | cell signaling |
| $\beta$-tubulin | mouse | IB | 1:10000 | T4026 | Sigma |
| $\beta$-catenin | mouse | IB | 1:1000 | 610153 | BD biosciences |
| F4/80 | rat | IHC | 1:100 | 14-4801-82 | ebioscience |
| Gapdh | mouse | IB | 1:10000 | ab9484 | Abcam |
| Cleaved Caspase 3 (Asp175) | rabbit | IHC | 1:300 | 9661 | cell signaling |
| HNF4$\alpha$ | goat | IHC | 1:1000 | sc-6556 | Santa Cruz |
| Gst1 | rabbit | IHC | 1:500 | ab16802 | Abcam |
| Nrf2 | rabbit | IB | 1:1000 | 12721 | Cell signaling |
| Epcam | rat | IF | 1:100 | 130-102-033 | ebiosciences |
| Cd133 | rat | IF | 1:100 | 11-1331-82 | ebiosciences |
| Cd44 | rat | IF | 1:100 | 550538 | BD Pharmingen |
| LC3 | rabbit | IB | 1:1000 | ab48394 | Abcam |
| Atg5 | rabbit | IB | 1:1000 | 2630 | Cell signaling |
| Nucleoporin p62 | mouse | IB | 1:1000 | 610497 | BD Biosciences |

**Supplementary Table 2. qRT-PCR primer sequences.**

| Gene | species | Forward Primer | Reverse Primer |
|---|---|---|---|
| *Gapdh* | mouse | CAATGACCCCTTCATTGACC | GATCTCGCTCCTGGAAGATG |
| *Afp* | mouse | AGTTTCCAGAACCTGCCGAG | ACCTTGTCGTACTGAGCAGC |
| *Areg* | mouse | TTGCTGCTGGTCTTAGGCTC | TGGTCCCCAGAAAGCGATTC |
| *Birc5* | mouse | TGCAAAGGAGACCAACAACA | GGCATGTCACTCAGGTCCAA |
| *Ctgf* | mouse | AGAACTGTGTACGGAGCGTG | GTGCACCATCTTTGGCAGTG |
| *Gli2* | mouse | GGGCATCCTCTCTGCTGTTT | CCCTCTTTGGGCGTGTACTT |
| *Itgb2* | mouse | GCTTTGGGTCGTTTGTGGAC | TGCCGACCTCTGTCTGAAAC |
| *Atg7* | mouse | TGGCTGCTACTTCTGCAATGAT | CAGGACAGAGACCATCAGCTCC |
| *Pcna* | mouse | ATCCTGAAGAAGGTGCTGGAGGCT | ACGAGTCCATGCTCTGCAGGTTCA |
| *Cyr61* | mouse | AGAGGCTTCCTGTCTTTGGC | CCAAGACGTGGTCTGAACGA |
| *Albumin* | mouse | CCCACTAGCCTCTGGCAAAA | ACACACCCCTGGAAAAAGCA |
| *18s* | mouse | GCAATTATTCCCCATGAACG | GGGACTTAATCAACGCAAGC |
| *Axin2* | mouse | CCTGACCAAACAGACGACGA | CACCTCTGCTGCCACAAAAC |
| *Yap* | mouse | CAGAGCCCACAGGGAGGCGT | GCTGCGCAGAGCTAATTCCTGACA |
| *Taz* | mouse | CAGCTCCACTTTCGGCCCGC | TTATCCCCGGAACCGGCCTC |
| *Cd133* | mouse | TCAGTTGATTCCAAGGAGATTGCCC | GCAGCCCACCAGAGGCATGA |
| *Cd44* | mouse | TCAGGAGCCCACAACGAG | GCTCCCAGCCTGTTGGGTT |
| *Epcam* | mouse | TACGACCCCGACTGCGACGA | CGGACTCCGGCGGTGTTGAC |
| *Tbp* | mouse | CACCCCCTTGTACCCTTCAC | CAGTTGTCCGTGGCTCTCTT |
| *$\beta$-actin* | mouse | TGATCCACATCTGCTGGA | GAAGAGCTACGAGCTGCC |
