# Supplementary material for: Autophagy is a gatekeeper of hepatic differentiation and carcinogenesis by controlling the degradation of Yap
Source: Nat Commun. 2018 Nov 23;9:4962. doi: 10.1038/s41467-018-07338-z (PMC6251897; doi:10.1038/s41467-018-07338-z)
Supplement: Supplementary file 3 — Reporting Summary [file 41467_2018_7338_MOESM3_ESM.pdf]

nature research

Corresponding author(s): Youngmin A. Lee

Reporting Summary

Nature Research wishes to improve the reproducibility of the work that we publish. This form provides structure for consistency and transparency in reporting. For further information on Nature Research policies, see Authors & Referees and the Editorial Policy Checklist.

Statistical parameters

When statistical analyses are reported, confirm that the following items are present in the relevant location (e.g. figure legend, table legend, main text, or Methods section).

n/a	Confirmed	
☐	☒	The exact sample size (n) for each experimental group/condition, given as a discrete number and unit of measurement
☐	☒	An indication of whether measurements were taken from distinct samples or whether the same sample was measured repeatedly
☐	☒	The statistical test(s) used AND whether they are one- or two-sided<br>*Only common tests should be described solely by name; describe more complex techniques in the Methods section.*
☒	☐	A description of all covariates tested
☐	☒	A description of any assumptions or corrections, such as tests of normality and adjustment for multiple comparisons
☐	☒	A full description of the statistics including central tendency (e.g. means) or other basic estimates (e.g. regression coefficient) AND variation (e.g. standard deviation) or associated estimates of uncertainty (e.g. confidence intervals)
☐	☒	For null hypothesis testing, the test statistic (e.g. F, t, r) with confidence intervals, effect sizes, degrees of freedom and P value noted<br>*Give P values as exact values whenever suitable.*
☒	☐	For Bayesian analysis, information on the choice of priors and Markov chain Monte Carlo settings
☒	☐	For hierarchical and complex designs, identification of the appropriate level for tests and full reporting of outcomes
☒	☐	Estimates of effect sizes (e.g. Cohen's d, Pearson's r), indicating how they were calculated
☐	☒	Clearly defined error bars<br>*State explicitly what error bars represent (e.g. SD, SE, CI)*

Our web collection on statistics for biologists may be useful.

Software and code

Policy information about availability of computer code

Data collection	Microsoft Excel for Mac (v16.17), Zeiss ZenBLUE, LightCycler (R) 480 software (v1.5.1.62 SP3), Veritas 1.9.2, Gen5 (v2.09),
Data analysis	Microsoft Excel, ImageJ (JACoP v.2.0), Prism 7, GSEA Desktop v3.0, MSigDB v6.0, Adobe Photoshop CC2015, Adobe Ilustrator CC 2015, Microsoft Powerpoint for Mac (v16.17).

For manuscripts utilizing custom algorithms or software that are central to the research but not yet described in published literature, software must be made available to editors/reviewers upon request. We strongly encourage code deposition in a community repository (e.g. GitHub). See the Nature Research guidelines for submitting code & software for further information.

Data

Policy information about availability of data

All manuscripts must include a data availability statement. This statement should provide the following information, where applicable:

- Accession codes, unique identifiers, or web links for publicly available datasets
- A list of figures that have associated raw data
- A description of any restrictions on data availability

The Gene Expression Omnibus accession number for the Atg7-ko transcriptome profiles reported in this paper is GSE67676. The Gene Expression Omnibus

accession number for 72 NAFLD-affected livers is GSE49541. Genome-wide transcriptome profiles of 374 human HCC tissues were obtained from The Cancer Genome Atlas data portal (https://gdc.cancer.gov).

Field-specific reporting

Please select the best fit for your research. If you are not sure, read the appropriate sections before making your selection.

☒ Life sciences ☐ Behavioural & social sciences ☐ Ecological, evolutionary & environmental sciences

For a reference copy of the document with all sections, see nature.com/authors/policies/ReportingSummary-flat.pdf

Life sciences study design

All studies must disclose on these points even when the disclosure is negative.

Sample size	In animal studies, average sample sizes of 5-10 animals per group were deemed representative. No statistical method had been used to predetermine sample size.
Data exclusions	no datapoints or animals were excluded in the analysis
Replication	Depending on the experiment, biological replicates were included (e.g. collection of cell lysates from consecutive cell passages of stable KO clones) or and each experiment was repeated at least twice. Technical replicates ensured to minimize variability e.g. Luciferase assays or qPCRs. All experiments were repeated at least twice.
Randomization	Animals were allocated to experimental groups depending on their genotype, both male and females were included in all studies.
Blinding	Investigators were blinded to group allocation during data collection and data analysis. Only after assessment and collection of all data points was the data organized according to groups.

Reporting for specific materials, systems and methods

Materials & experimental systems

n/a	Involved in the study	
☐	☒	Unique biological materials
☐	☒	Antibodies
☐	☒	Eukaryotic cell lines
☒	☐	Palaeontology
☐	☒	Animals and other organisms
☐	☒	Human research participants

Methods

n/a	Involved in the study	
☒	☐	ChIP-seq
☒	☐	Flow cytometry
☒	☐	MRI-based neuroimaging

Unique biological materials

Policy information about availability of materials

Obtaining unique materials	Tead4-Luciferase reporter was obtained from Dr. Fernando Camargo and is not commercially available.

Antibodies

Antibodies used	All antibodies used in the study have been listed in Methods, Table 1. Manufacturer, catalog number, species, dilution and application used are also included in this table.
Validation	All antibodies used in this study are commercially available. We have included an attachment detaling the requested information in a separate document named "Addendum to Reporting Summary Lee et al".

Eukaryotic cell lines

Policy information about cell lines

Cell line source(s)	AML12 cells: ATCC, THLE5B cells, shared by Yujin Hoshida (coauthor)
Authentication	THLE5B were analyzed by short tandem repeat profiling.
Mycoplasma contamination	all cell lines were tested for mycoplasma contaminaten.
Commonly misidentified lines (See ICLAC register)	Neither AML12 nor THLE5B are listed in the ICLAC register (v8.0).

Animals and other organisms

Policy information about studies involving animals; ARRIVE guidelines recommended for reporting animal research

Laboratory animals	Mice, C57/BL6, Albumin-CRE, m/f, between 6 weeks and 14 months.<br>Mice, C57/BL6, Albumin-CRE/Atg7flox/flox, m/f, between 6 weeks and 14 months.<br>Mice, C57/BL6, Atg7flox/flox, m/f, between 6 weeks and 14 months.<br>Mice, C57/BL6, ERT2-Albumin-CRE/Atg7flox/flox, m/f, between 6 weeks and 14 months.<br>Mice, C57/BL6, ERT2-Albumin-CRE/Atg7flox/flox/Yapflox/wt, m/f, between 6 weeks and 14 months.<br>Mice, C57/BL6, ERT2-Albumin-CRE/Atg7flox/flox/Yapflox/flox, m/f, between 6 weeks and 14 months.<br>Mice, C57/BL6, Atg7flox/flox/Yapflox/wt, m/f, between 6 weeks and 14 months.<br>Mice, C57/BL6, Atg7flox/flox/Yapflox/flox, m/f, between 6 weeks and 14 months.<br>Mice, C57/Bl6, Nrf2-/-, m/f, 8 weeks<br>Mice, C57/BL6, Albumin-CRE/Atg7flox/flox/Nrf2-/- , m/f, 8 weeks
Wild animals	na
Field-collected samples	na

Human research participants

Policy information about studies involving human research participants

Population characteristics	Human liver tissues from patients undergoing liver resection for liver cancer were obtained from the Biorepository and Pathology CORE of Icahn School of Medicine at Mount Sinai. No further details with regards to gender, genotype, past diagnoses or diagnoses other than liver cancer are known.
Recruitment	Patients are consented by the Biorepository and Pathology CORE of Icahn School of Medicine at Mount Sinai. Thus, no direct contact between patients and investigators exist eliminating any self-selection bias.
