# Supplementary material for: Autophagy is a gatekeeper of hepatic differentiation and carcinogenesis by controlling the degradation of Yap
Source: Nat Commun. 2018 Nov 23;9:4962. doi: 10.1038/s41467-018-07338-z (PMC6251897; doi:10.1038/s41467-018-07338-z)
Supplement: Supplementary file 4 — Addendum to Reporting Summary [file 41467_2018_7338_MOESM4_ESM.pdf]

Addendum to Reporting Summary Lee et al final.

Describe the validation of each primary antibody for the species and application noting any validation statements on the manufacturers website, relevant citations, antibody profiles in online databases and data provided in the manuscript.

Antibody	species	application	dilution	cat. Number	manufacturer	Validation statement on man. website	Relevant citations	Data provided in the manuscript	Other means
Yap	rabbit	IB	1:1000	4912	cell signaling	YAP Antibody detects endogenous levels of total YAP protein. Western blot analysis on extracts from NIH/3T3, C6, MDA-MB-231	1. Zhu, B el al. Sci Rep. 2018 Aug 29;8(1):13025. 2. Hsu, PC et al. J Cell Mol Med. 2018 Jun;22(6):3073-3085.		Testing of transiently Yap transfected THLE5B cells (addgene 19057, addgene 19045). Data not included in manuscript but available upon request
		IHC	1:500	4912	cell signaling	IHC of paraffin-embedded human breast carcinoma, human prostate carcinoma showing cytoplasmic and nuclear localization, using YAP Antibody.	1. Shen, J et al. Journal of Experimental & Clinical Cancer Research (2018) 37:175 2. Zhu, B el al. Sci Rep. 2018 Aug 29;8(1):13025		
Yap	rabbit	IB	1:1000	14074	cell signaling	YAP (D8H1X) XP® Rabbit mAb recognizes endogenous levels of total YAP protein. Western blot analysis of extracts from various cell lines using YAP (D8H1X) XP® Rabbit mAb	1. Mou, K et al. Oncology Reports 40: 2056-2066, 2018. 2. Kato, K et al. Nat Commun. 2018 Jun 22;9(1):2448. 2. Kato, K et al. Nat Commun.		Testing of transiently Yap transfected THLE5B cells (addgene 19057, addgene 19045). Data not included in manuscript

							2018 Jun 22;9(1):2448.		but available upon request.
		IHC	1:500	14074	cell signaling	IHC of paraffin-embedded human breast adenocarcinoma, ovarian serous carcinoma,human benign prostatic hyperplasia, PANC1, RL-7 cell pellets.	1. Chibly, AM et al. Sci Rep. 2018 Apr 20;8(1):6347.		
Taz	rabbit	IB	1:1000	HPA007415	Sigma	Western blot analysis of EFO-21 cells transfected with control siRNA, target specific probe #1 and #2 for enhanced validation of RNAi knockdown. Loading control was equal.	1. Di Agostino, S et al. EMBO Rep. 2016 Feb;17(2):188-201. 1. Gruber, R et al. Gastroenterology. 2016 Sep;151(3):526-39.	band at expected molecular weight in liver lysates. Data included in manuscript	band at expected molecular weight in liver lysates. Data included in manuscript
		IHC	1:500	HPA007415	Sigma	IHC staining of different human tissues such as placenta, kidney, renal cancer, colon, and skeletal muscle.	1. Slemmons, KK et al. PLoS One. 2015 Oct 23;10(10): e0140781. 2. Liu, F et al. Am J Physiol Lung Cell Mol Physiol. 2015 Feb 15;308(4): L344-57.		
Ki67	rabbit	IHC	1:10000	15580	abcam	Ab15580 is batch tested in ICC/IHC. IHC image ab15580 staining in mouse spleen FFPE tissue section.	1. Boddaert, J et al. PLoS One. 2018 Jan 17;13(1):e0186937. 2. Smith, MD et al. Dev Cell. 2018 Jan		Highly specific nuclear staining in mouse liver tissue. IHC tested in mouse

							22;44(2):217-232.e11.		models with high hepatic regeneration (partial hepatectomy). Good correlation to BrdU staining and PCNA (unpublished results, not in manuscript but available upon request)
p62/SQSTM1	rabbit	IB	1:4000	PM045	MBL	Antibody is validated for multiple applications (WB, IHC, ICC and IP) and is widely cited, including in research papers with more than 200 citations (Waguri S, Komatsu M. Biochemical and morphological detection of inclusion bodies in autophagy-deficient mice. Methods Enzymol. 453, 181-96 (2009)). This is a polyclonal antibody of 100 ul that is raised in rabbit and is reactive with human, hamster, mouse, rat.	1. Frankel, LB et al. EMBO J. 2011 Sep 13;30(22):4628-41.<br>2. Lee, EJ & Tournier, C. Autophagy. 2011 Jul;7(7):689-95. Epub 2011 July 1.<br>3. Waguri, S & Komatsu, M. Methods Enzymol. 453, 181-96 (2009).	Band at expected molecular weight. Increase in positive controls (livers with autophagy impairment/Atg7 ko livers).	
		IHC	1:4000	PM045	MBL	Antibody is validated for multiple	1. Waguri, S & Komatsu, M. Methods	Very typical staining in liver tissue of Atg7 KO mice, identical to	

						applications (WB, IHC, ICC and IP) and is widely cited, including in research papers with more than 200 citations (Waguri S, Komatsu M. Biochemical and morphological detection of inclusion bodies in autophagy-deficient mice. Methods Enzymol. 453, 181-96 (2009)). This is a polyclonal antibody of 100 ul that is raised in rabbit and is reactive with human, hamster, mouse, rat.	Enzymol. 453, 181-96 (2009).	previously published papers (Inami et al, 2011 J Cell Biol, 193(2):275.)	
Atg7	rabbit	IB	1:1000	A2856	Sigma	Anti-Atg7 recognizes human, mouse, and rat Atg7 by immunoblotting (~75 kDa), immunoprecipitation and immunohistochemistry. Western blot analysis of whole mouse 3T3 cell extract was separated on SDS-PAGE and blotted with Rabbit Anti-ATG7.	1. Michiels, CF et al. Am J Physiol Heart Circ Physiol. 2015 Mar 15;308(6):H557-67.	Band at exp ected molecular weight in IB. Loss of band in Atg7 KO mice. Data included in the manuscript.	
Yap/Taz	rabbit	IB	1:1000	8418	cell signaling	Western blot analysis of extracts from various cell lines using	1. Hernandez, C et al. J Clin Invest. 2018 Jun		Bands at expected molecular weights.

						YAP/TAZ (D24E4) Rabbit mAb.	1;128(6):2436-2451.		Data available on request.
P-Yap (S127)	rabbit	IB	1:1000	4911	cell signaling	Western blot analysis of extracts from MDA-MB-468 cells, untreated or treated with wortmannin and LY294002, using Phospho-YAP (Ser127) Antibody. The phospho-specificity of the antibody was validated by treating the membrane with (+) or without (-) calf intestinal phosphatase (CIP) after western transfer.	1. Zhu, B et al. Sci Rep. 2018 Aug 29;8(1):13025.<br>2. Yao, F et al. Nat Commun. 2018 Jun 11;9(1):2269.	Band at expected molecular weight. Data included in the manuscript.	
Beta-tubulin	mouse	IB	1:10000	T4026	Sigma	It localizes b-tubulin in human, bovine, rat, mouse, sea urchin, and plant b-tubulin. It recognizes all five isoforms of b-tubulin (b1-b5), and may be used in immunoblotting, 2-dimensional electrophoresis, sperm motility, and immunohistochemistry. It is immunospecific for tubulin as determined by indirect immunofluorescent staining and	1. Lecat, A et al. J Biol Chem. 2012 Aug 24;287(35):29213-26.<br>2. Tarazona, R et al. J Immunol. 2000 Dec 15;165(12):6776-82.	Band at expected molecular weight. Data included in the manuscript.	.

						immunoblotting procedures.			
Beta-catenin	mouse	IHC	1:1000	610153	BD biosciences	Antibody is routinely tested by western blot. IHC, IF and IP were tested during development.	1. Tateishi, K et al. J Cell Biol. 2001; 155(4):571-579.		
F4/80	rat	IHC	1:100	14-4801-82	ebioscience	The BM8 antibody has been reported for use in flow cytometric analysis, immunohistochemical staining of frozen tissue sections, and immunohistochemical staining of paraffin embedded tissue sections. The BM8 antibody has been tested by flow cytometric analysis of mouse spleen or bone marrow cells. The BM8 antibody has been tested by immunohistochemistry of formalin-fixed paraffin embedded mouse tissue.	1. Diaconu, I et al. Cancer Res. 2012 May 1;72(9):2327-38.<br>2. Ying, W et al. J Clin Invest. 2015 Nov 2;125(11):4149-59.		Validated antibody in flow analysis with other markers for Kupffer cells
Gapdh	mouse	IB	1:10000	ab9484	Abcam	Our Abpromise guarantee covers the use of **ab9484** in the following tested applications: WB, IHC-P, Flow cytometry.	1. Krysiak J et al. Nat Commun. 2018 Jan 17;9(1):262.<br>2. Zhang, XL et al. Oncol Lett. 2018 Apr;15(4):6003-6008.		Band at expected molecular weight

Cleaved Caspase 3 (Asp175)	rabbit	IHC	1:300	9661	cell signaling	Western blot analysis of extracts from HeLa, NIH/3T3 and C6 cells untreated, staurosporine-treated (3hrs, 1 µM in vivo) or cytochrome c-treated (1hr, 0.25 mg/ml in vitro). Immunohistochemical analysis of paraffin-embedded mouse embryo, using Cleaved Caspase-3 (Asp175) Antibody preincubated with control peptide.	1. Beug St, et al. Mol Ther Oncolytics. 2018 Jun 21;10:28-39.<br>2. Meyer, A et al. Front Integr Neurosci. 2018 Aug 14;12:35.		
HNF4alpha	goat	IHC	1:1000	sc-6556	Santa Cruz	Recommended for detection of HNF-4α of mouse, rat and human origin by WB, IP, IF and ELISA; also reactive with additional species, including and equine, canine, bovine and porcine.	1. Alpern, D et al. Elife. 2014 Sep 10;3:e03613.		Validated by immunoblotting: Band at expected molecular weight. In IHC typical nuclear staining of hepatocytes (can be identified without co-staining).
Gst1	rabbit	IHC	1:500	Ab16802	Abcam	Our Abpromise guarantee covers the use of ab16802 in the following tested applications: ICC/IF, IHC-Fr, WB, In-cell ELISA,and IHC-FoFr.	1. Orr, AG et al. Nat Neurosci. 2015 Mar;18(3):423-34.<br>2. Burkovetskaya, M et al. PLoS One. 2014 Apr		Validation by IHC only. Typical strong staining in centrilobular region

							15;9(4):e95023 .		
Nrf2	rabbit	IB	1:1000	12721	Cell signaling	Western blot analysis of extracts from MEF wt and U-2 OS cells, untreated (-) or treated with MG-132 #2194 (10 µM, 10 hr; +).	1. Xue, X et al. Proc Natl Acad Sci U S A. 2017 Nov 7;114(45):E9608-E9617.<br>1. Liu, S et al. Oncotarget. 2018 Jan 3;9(14):11528-11540.		MG132 treatment to increase Nrf2 expression in cultured cells. Data available upon request.
Epcam	rat	IF	1:100	130-102-033	Miltenyi Biotec	Immunofluorescent microscopic images of fixed mouse embryonic stem cells (HM1) cultured on feeder cells: cells were fixed in PFA and stained with CD326 (EpCAM)-Biotin overnight at 4 °C. Detection of CD326 (EpCAM)-Biotin was performed with a secondary antibody.			Data validated by flow analysis of dissociated single cell liver. Data not included but available upon request.
Cd133	rat	IF	1:100	11-1331-82	ebiosciences	Published figure using CD133 (Prominin-1) monoclonal antibody (Product # 11-1331-82).	1. Miller, Te et al. PLoS One. 2014 Dec 10;9(12):e114433.		Data validated by flow analysis of dissociated single cell liver. Data not included but available upon request.
Cd44	rat	IF	1:100	550538	BD Pharmingen	The IM7 antibody specifically			Data validated by

						recognizes an epitope on both alloantigens and all isoforms of the CD44 glycoprotein (Pgp-1, Ly-24). Antibody routinely tested in Flow cytometry. The IM7 antibody specific for mouse CD44 is recommended to test for immunohistochemical staining of formalin-fixed paraffin and acetone-fixed frozen sections.			flow analysis of dissociated single cell liver. Data not included but available upon request.
LC3	rabbit	IB	1:1000	ab48394	Abcam	Our Abpromise guarantee covers the use of ab48394 in the following tested applications: ICC/IF, IP, IHC-P, WB, IHC-Fr.	1. Magalhaes, J et al. Sci Rep. 2018 Jan 23;8(1):1385.	Validation by IB. expected molecular weight. Alteration in response to inhibition of autophagy by genetic deletion. Use of established lysosomal or autophagy inhibitors (3-Methyladenine, Leupepting/Aminochloride with alteration in LC3 I, II ratio)	
Atg5	rabbit	IB	1:1000	2630	Cell signaling	Western blot analysis of extracts from various cell lines.	1. Chai, K et al. Oncotarget. 2018 May 25;9(40):26130-26143.	Band at expected molecular weight. Decrease with shATG5 in cell lysates. Data is included in manuscript	
Nucleoporin p62	mouse	IB	1:1000	610497	BD Biosciences	Antibody is routinely tested in western blot. Immunoprecipitation and	1. Shah , M et al. J Biol Chem. 2002; 277(47):45662-45669.	Bands at expected molecular weight in cytoplasmic and nuclear fractionation of cell lysates. Data	

						immunofluorescence were tested during development.		included in manuscript.	
